# Targeted therapy of papillary craniopharyngioma

**DOI:** 10.1007/s12032-025-02920-0

**Published:** 2025-07-22

**Authors:** Ning Luo, Yi Lin, Tao Hong, Zhixiong Lin

**Affiliations:** 1 Department of Neurosurgery, Sanbo Brain Hospital, Capital Medical University, Xiangshanyikesong 50#, HaiDian District, Beijing, 100093 China; 2 Department of Neurosurgery, The First Affiliated Hospital, Jiangxi Medical College, Nanchang University, Nanchang, 330200 Jiangxi China

**Keywords:** Craniopharyngioma, Papillary craniopharyngioma, Molecular biology, Targeted therapy, Comprehensive treatment

## Abstract

This review provide a comprehensive overview of the molecular biology and therapeutic advances regarding papillary craniopharyngiomas (PCP), with a particular focus on the pivotal role of the BRAF V600E mutation in its pathogenesis. Histopathologically, PCPs are characterized by stratified squamous epithelium and are frequently associated with the BRAF V600E mutation. This mutation activates the MAPK/ERK signaling pathway, which drives tumor development and progression. The identification of this pathway has led to significant progress in targeted therapies, specifically with the use of BRAF and MEK inhibitors, which have demonstrated remarkable efficacy in clinical trials. These inhibitors can effectively reduce tumor size and improve clinical outcomes for patients. However, despite these advancements, there are challenges such as the potential for resistance to these therapies and the management of long-term side effects. Consequently, a multidisciplinary approach that combines surgical resection, radiation therapy, and targeted therapy is often recommended to enhance treatment efficacy although minimizing adverse effects. In addition to adult cases, this review also addresses rare instances of pediatric PCP. Although these cases are infrequent, their molecular characteristics closely resemble those of adult PCP, suggesting that similar therapeutic approaches might be applicable. Looking ahead, future research should focus on optimizing treatment regimens, understanding the interactions within the tumor's immune microenvironment, and identifying novel therapeutic targets. These efforts are crucial for enhancing precision medicine strategies for PCP patients, ultimately improving their quality of life and long-term prognosis. Overall, continued exploration in this field holds promise for more effective and tailored treatment options.

## Introduction

Craniopharyngiomas (CP) are epithelial tumors of the sellar and parasellar regions, originating from the ectodermal remnants of the oral cavity and Rathke’s pouch, adjacent to critical structures, such as the hypothalamic–pituitary axis and optic nerves. Craniopharyngiomas (CPs) comprise 1.2–4.6% of all intracranial tumors, with an estimated incidence of 1.3 cases per million person-years [1]. Although histologically benign, CPs frequently result in severe visual impairments, endocrine disturbances, cognitive deficits, and personality alterations due to their location near essential structures. These clinical manifestations are not solely attributable to tumor progression but are also strongly associated with the effects of surgical and radiotherapeutic interventions [2, 3]. The chronic complications arising from these treatments represent the primary contributors to reduced quality of life and social impairment in affected individuals [4]. Historically, CP was divided into two subtypes: adamantinomatous craniopharyngiomas (ACP) and papillary craniopharyngiomas (PCP) [5–9]. The 5th edition of the WHO Classification of Tumors of the Central Nervous System (WHO CNS5) reclassified CP based on histopathological and genetic differences, considering ACP and PCP as distinct tumor entities rather than subtypes [10]. ACP is histologically characterized by clusters of “wet” keratin, frequent calcifications, and cystic changes, with CTNNB1 mutations being the primary driver [11]. PCP, on the other hand, consists of stratified squamous epithelium, with BRAF V600E mutations identified as the primary molecular alteration [12].

PCP is a rare but challenging central nervous system tumor, predominantly affecting adults. Despite the advances in surgical and radiation therapies, the treatment of PCP remains complex due to its anatomical location and high recurrence rates, thus, novel therapeutic strategies are crucial. In recent years, breakthroughs have been made in understanding the molecular biology of PCP, particularly regarding the BRAF V600E mutation, leading to significant advances in targeted therapies and affirming its status as a distinct tumor entity. So it is essential to single it out for focused attention and discussion. Although PCP was previously thought to be exclusive to adults, the identification of BRAF V600E mutations has confirmed the existence of pediatric papillary craniopharyngiomas (PPCP) [13]. Although PCP cases predominantly occur in adults, the increasing reports of pediatric PCP highlight the potential risk of misdiagnosis in this population. Therefore, in-depth research on PCP can not only advance the development of more precise, individualized treatment plans but also enhance our overall understanding of the mechanisms of rare intracranial tumors. This review aims to summarize the molecular characteristics of PCP, the discovery of PPCP, the latest advances in targeted therapy, and the clinical application of various treatment strategies.

## Histopathological characteristics of PCP

**Fig. 1:**
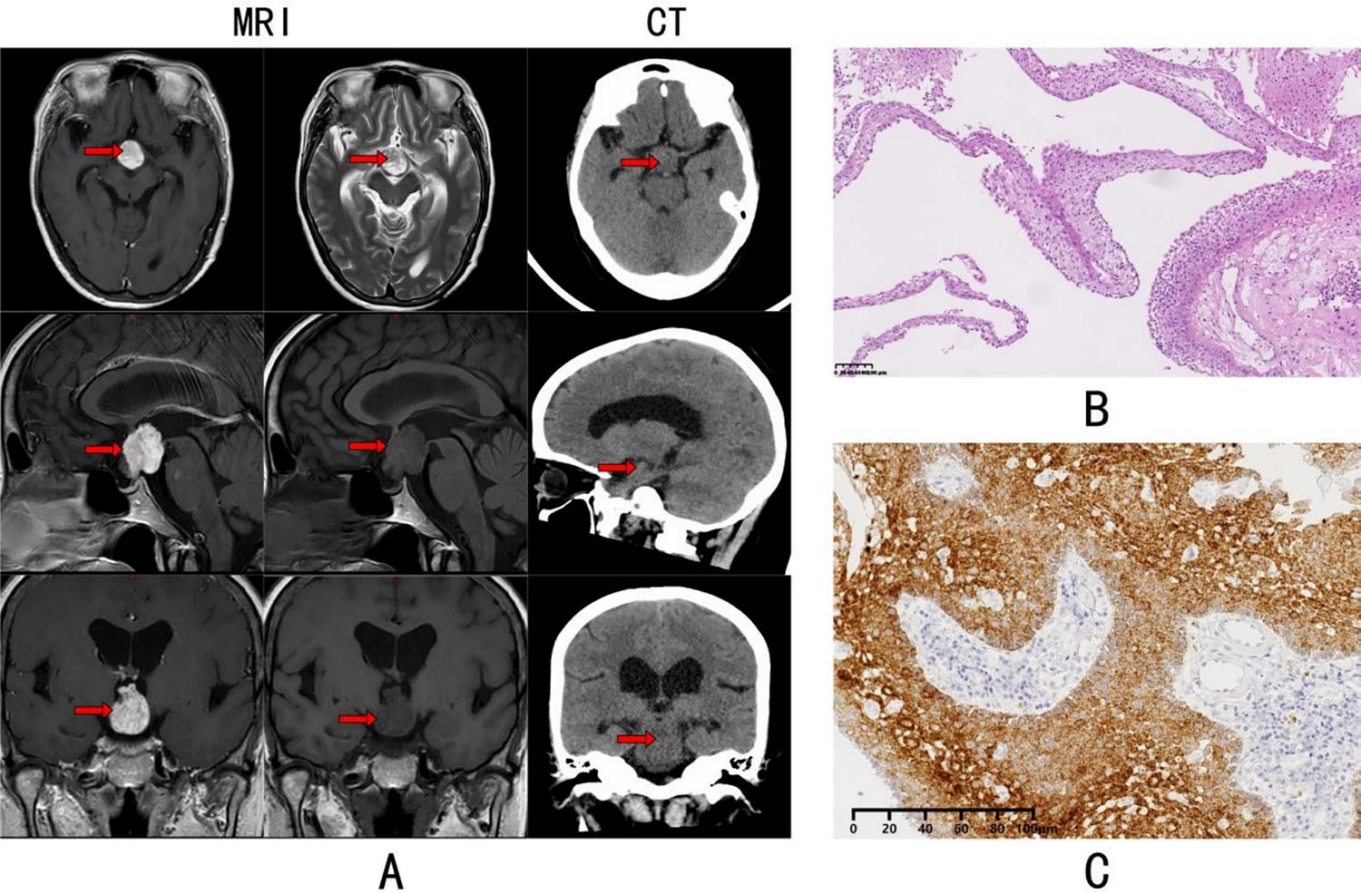
Radiological and histopathological characteristics of papillary craniopharyngioma. **A** MRI and CT images of the sellar region reveal a well-circumscribed mass (indicated by the red arrow). The mass appears isointense to mildly hyperintense on T1-weighted MRI, hyperintense on T2-weighted MRI, and exhibits homogeneous enhancement post contrast. The corresponding CT images show no calcification within the mass. **B** H&E-stained histological section of the tumor displays papillary structures with a fibrovascular core, lined by a single layer of epithelial cells. **C** Immunohistochemical staining of the tumor reveals diffuse positive staining for the BRAFV600E mutation in the tumor cells. MRI, magnetic resonance imaging; CT, computed tomography; H&E, hematoxylin and eosin staining; BRAFV600E, B-Raf proto-oncogene V600E mutation

PCP is histologically composed of stratified squamous epithelium, where epithelial protrusions infiltrate surrounding tissues as epithelial cords. The cell layers are compact, lacking areas of stellate cells or whorled structures, and there is an absence of wet keratin, with little to no calcification or cystic degeneration. Immunohistochemistry often reveals positive BRAF V600E expression in the cytoplasm of tumor cells. Compared with adult PCP, although systematic comparative studies are lacking, preliminary data suggest that PPCP often exhibits intense inflammatory infiltration, sometimes even resembling abscess formation [13, 14]. These inflammatory changes can lead to misdiagnosis [13] and should be carefully considered, underscoring the need for a systematic comparison of clinical presentation, imaging features, molecular biology, and particularly the immune microenvironment between adult PCP and PPCP to avoid misdiagnosis and inappropriate treatment (Fig. 1).

## Molecular characteristics of PCP

**Fig. 2:**
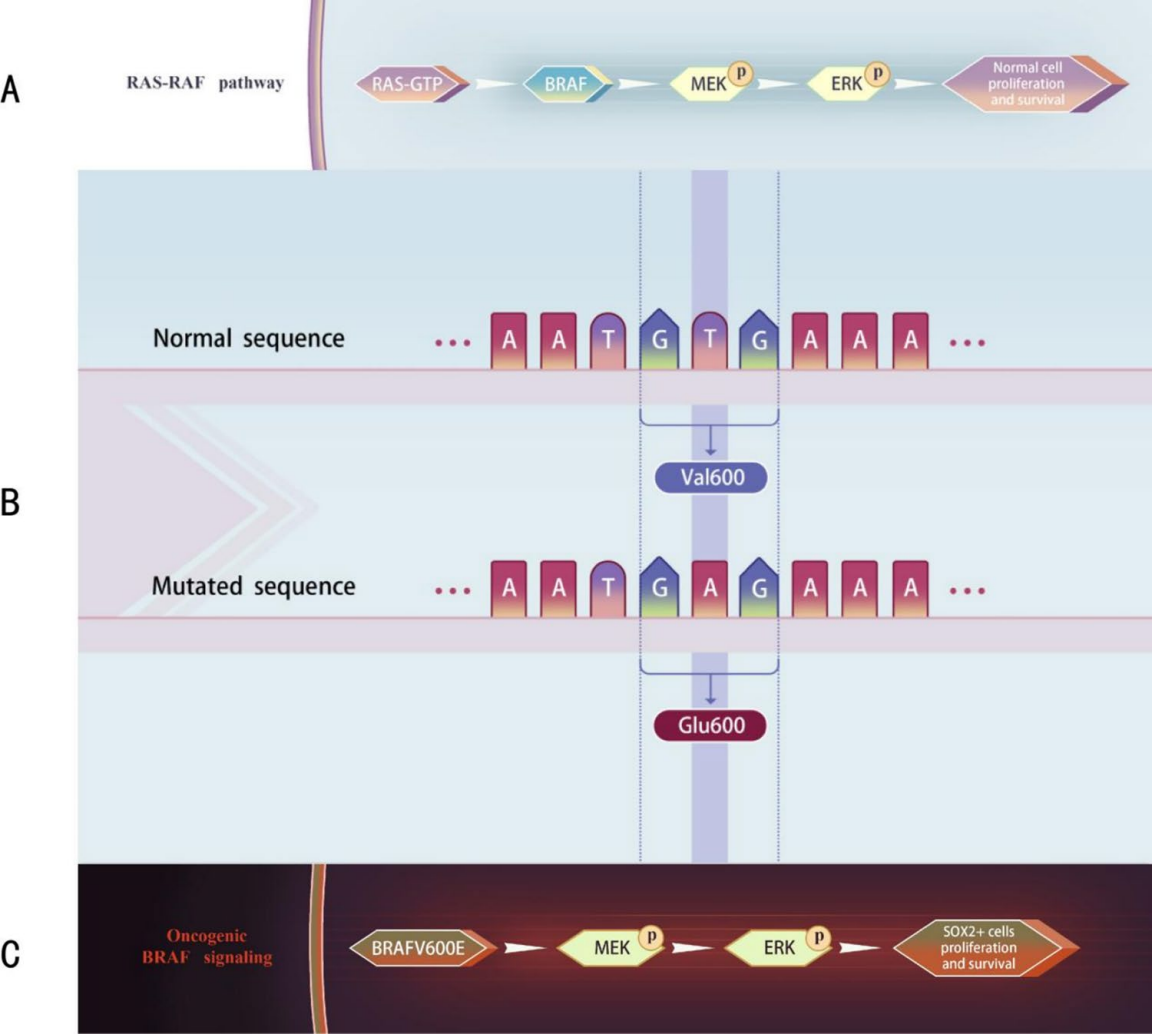
The BRAF V600E mutation and its impact on the RAS–RAF pathway. **A** The RAS–RAF signaling pathway under normal conditions. Binding of RAS-GTP activates BRAF, leading to the phosphorylation of MEK and ERK, which ultimately promotes normal cell proliferation and survival. **B** Schematic of the normal and mutated sequences of the BRAF gene. The normal sequence encodes valine at position 600 (Val600), whereas the mutated sequence shows a substitution of thymine (T) by adenine (A), resulting in the encoding of glutamic acid (Glu600) instead of valine. **C** The oncogenic BRAFV600E signaling pathway. The BRAF^V600E mutation results in constitutive activation of BRAF, leading to continuous phosphorylation of MEK and ERK. This aberrant signaling promotes the proliferation and survival of SOX2 + cells, driving tumorigenesis. RAS-GTP, GTP-bound RAS; BRAF, B-Raf proto-oncogene; MEK, mitogen-activated protein kinase kinase; ERK, extracellular signal-regulated kinase; SOX2, SRY-box transcription factor 2

Research has established that nearly all PCP tumors harbor the BRAF V600E mutation, which is recognized as the sole driver mutation for PCP [12, 15–17]. BRAF is a critical kinase in the MAPK signaling pathway. Under normal circumstances, BRAF is activated by RAS proteins and initiates a downstream cascade of signaling events. When external growth factors stimulate RAS proteins, they convert to their active form (RAS-GTP), which binds to and activates BRAF by inducing conformational changes. Activated BRAF then phosphorylates and activates MEK (MAPK/ERK kinase), which in turn phosphorylates and activates ERK (extracellular signal-regulated kinase), triggering signaling pathways that promote cell proliferation, differentiation, and survival [18, 19] (Fig. 2A).

However, the BRAF V600E mutation (substitution of valine by glutamic acid at position 600) alters the structure of the BRAF protein (Fig. 2B), resulting in a conformational change that significantly enhances its kinase activity. This mutant form of BRAF can activate itself without requiring activation by RAS-GTP, remaining in a constitutively active state (Fig. 2C). Specifically, the BRAF V600E mutation introduces a negatively charged glutamic acid, mimicking the phosphorylation state of activated BRAF. This “pseudo-activation” enables the mutant BRAF protein to form homodimers or heterodimers with other activated BRAF molecules, autonomously phosphorylating and activating MEK (Fig. 2C). Once MEK is activated by BRAF V600E, it phosphorylates ERK at tyrosine and threonine residues, resulting in ERK activation. Activated ERK then translocates to the nucleus, where it phosphorylates transcription factors regulating the expression of downstream genes involved in cell cycle progression, proliferation, survival, angiogenesis, and inhibition of apoptosis [20–22].

This sustained activation of the MAPK/ERK signaling pathway is particularly evident in Sex Determining Region Y Box 2(SOX2) + stem cells. Using mouse model to investigate the activation of the MAPK/ERK pathway in embryonic pituitary tissues, researchers found a marked expansion of the SOX2 + cell population, occupying nearly three-quarters of the total cell population. In this model, over 90% of proliferative tumor cells were positive for SOX2 (a stem cell marker) and pERK1 + /ERK2 + (downstream mediators of BRAF and major effectors of MAPK). This suggests that MAPK pathway activation in SOX2 + cells grants them a proliferative advantage although impairing their differentiation potential. This combination of abnormal proliferation and differentiation defects indicates that SOX2 + cells may serve as the tumor-initiating cells for PCP [23].

Mu et al. [24] proposed a novel theory linking adult-onset PCPs to β2-tanycytes, specialized ependymoglial cells in the third ventricular floor (3VF). These cells reside in the median eminence (ME)—a region lacking a blood–brain barrier—and remain quiescent under physiological conditions but can proliferate in response to injury [25–28]. Experimental induction of BRAFV600E in Ras + β2-tanycytes of adult mice led to dedifferentiation and formation of PCP-like tumors, characterized by SOX2/SOX9 + stem-like cells in the tumor core and a peripheral Ki67 + proliferative zone [24]. This model aligns with the anatomical predilection of PCPs for the infundibulotuberal region, offering a compelling alternative origin for adult tumors that warrants further validation [29].

In recent years, increasing attention has been given to the tumor microenvironment and its role in tumor progression, leading to significant advances in understanding the molecular biology of the PCP microenvironment. In 2018, Coy et al. reported significant expression of PD-L1 in basal tumor cells, particularly in multilayered cells surrounding the fibrovascular stroma. This expression pattern may allow tumor cells to evade immune surveillance by modulating immune cell activity. Furthermore, PCP contains a large number of lymphocytes, including B cells and T cells, with some B cells also expressing PD-L1. Although CD8+ T cells are present in lower densities, their presence suggests some level of immune activation within the tumor microenvironment. PD-L1 expression was not found to be associated with tumor recurrence or prior radiation therapy, suggesting that PD-L1 may function as an inherent immune escape mechanism. Given the efficacy and tolerability of PD-1/PD-L1 inhibitors in other tumor types, these findings support the potential exploration of PD-1/PD-L1-targeted therapies in PCP [30].

Chen et al. [31] reported high expression of B7-H3 (CD276) in both ACP and PCP, which was associated with poor prognosis. Subsequent studies in 2022 demonstrated tumor suppression using antibody–drug conjugates in organoid models [32]. In another study from 2022, Zhao et al. reported significant complexity and diversity within the tumor immune microenvironment of PPCP. Immunohistochemical analyses revealed positive expression of several inflammatory cell markers, including MPO, CD3, CD20, CD38, CD68, and CD163. The presence of these immune cells suggests an active immune response within the tumor microenvironment, potentially contributing to tumor progression, invasion, and immune evasion, providing a rationale for future immunotherapeutic approaches [13].

Furthermore, Jia et al. [33] found that PCP was characterized by high levels of neutrophil infiltration, which may suppress immune microenvironment activity. Increased expression of IL-1A and IL-6 was positively correlated with hypothalamic invasion, suggesting their potential as therapeutic targets. Lin et al. reported that recurrent craniopharyngiomas (both PCP and ACP) exhibited increased M2 macrophages and PD-L1 expression, with a positive correlation between M2 macrophages and CD8+ T cells and PD-L1 expression. These findings indicate that PD-L1 plays a critical role in immune evasion, particularly through its association with the fibrovascular stroma in PCP, further supporting PD-1/PD-L1-targeted therapies and M2 macrophage modulation as potential treatment strategies [34].

In summary, current molecular studies on PCP have revealed several key mechanisms driving tumorigenesis and progression. The BRAF V600E mutation activates the MAPK/ERK pathway, driving abnormal proliferation and impaired differentiation of SOX2 + cells, which likely serve as the cellular basis for tumor initiation. The involvement of immune-regulatory molecules within the tumor microenvironment, such as PD-L1 and B7-H3, along with various immune cells and inflammatory factors, further complicates the tumor's immune evasion and invasive behavior. These findings not only deepen our understanding of PCP pathogenesis but also offer valuable insights into future therapeutic strategies, including targeted and immunotherapy approaches. However, further research is needed to clarify the link between the immune microenvironment and the abnormal MAPK/ERK pathway activation driven by BRAF V600E mutations in PCP.

## Conventional treatment of papillary craniopharyngiomas

In the past, discussions on the treatment of PCP were often included within the broader scope of craniopharyngiomas (CP), with little distinction made for PCP specifically. As PCP is a benign tumor histologically, complete surgical resection theoretically offers a cure. Matson et al. [35] proposed that the optimal treatment strategy for CP is to achieve as complete a resection as possible during the initial surgery. Similarly, Yasargil and Hoffman also advocated for gross total resection [36, 37]. Maira further emphasized that total tumor removal can avoid the risks and challenges of a second surgery [38]. The strong gliotic reaction between the tumor and normal brain tissue, characteristic of craniopharyngiomas, facilitates complete resection [39]. Compared with ACP, PCP is thought to exhibit less adhesion to hypothalamic structures, theoretically allowing for safer and more complete surgical resection. However, since most of the literature has reported ACP and PCP together, no robust comparative data is available to validate these differences.

Despite the shared goal among neurosurgeons of achieving gross total resection, the complex location of the tumor and its adherence to surrounding structures often makes aggressive resection risky, leading to significant postoperative complications. Therefore, radiation therapy has played a crucial role in the management of CP. The current consensus suggests that partial resection, followed by radiotherapy for residual tumor, can achieve outcomes similar to gross total resection in terms of long-term efficacy [40]. Unfortunately, as with surgical outcome assessments, the literature does not distinguish between PCP and ACP in terms of their radiation sensitivity, which is regrettable. However, some studies provide useful insights into radiotherapy techniques. For instance, stereotactic radiosurgery (SRS) has been shown to have advantages in treating CP [41–43]. Specific benefits of SRS include: (1) precise targeting of the tumor, minimizing damage to surrounding normal tissue and reducing the risk of complications. Studies have reported the use of smaller marginal doses (11.7 Gy) when treating small tumors (2.6 ml) with Gamma Knife, without inducing radiation injury. (2) SRS offers long-term efficacy and safety in CP management, with progression-free survival (PFS) rates of 76% at 5 and 10 years, and overall survival (OS) rates of 96% and 86%, respectively. Another study highlighted that fractionated stereotactic radiotherapy (FSRT) following endoscopic cyst aspiration and fenestration yielded good long-term tumor control. Future research specifically focused on PCP radiotherapy is warranted to obtain more precise data.

In addition to surgery and radiotherapy, bleomycin has been used in the treatment of CP, particularly in cystic and cyst-solid variants [44]. However, the literature does not differentiate between ACP and PCP. The standard method involves injecting 2–5 mg of bleomycin into the cyst twice or three times per week. Studies have found that the efficacy of cyst reduction ranges from 50 to 70% [45, 46]. Bleomycin helps separate vascular and neural structures, facilitating tumor resection. However, its neurotoxicity is significant, and any leakage of cyst fluid into the cerebrospinal fluid can be highly toxic, potentially leading to blindness or even death. Therefore, intralesional bleomycin chemotherapy is only administered when the cyst is sealed and there is no contrast leakage. Additionally, a multicenter prospective study found that intralesional α-interferon therapy was successful in some CP patients [47]. Nevertheless, none of these chemotherapy studies differentiate between histological subtypes, and given the predominantly solid imaging characteristics of PCP, it can be inferred that past chemotherapy efforts likely focused primarily on ACP.

## Targeted therapy for PCP

**Fig. 3:**
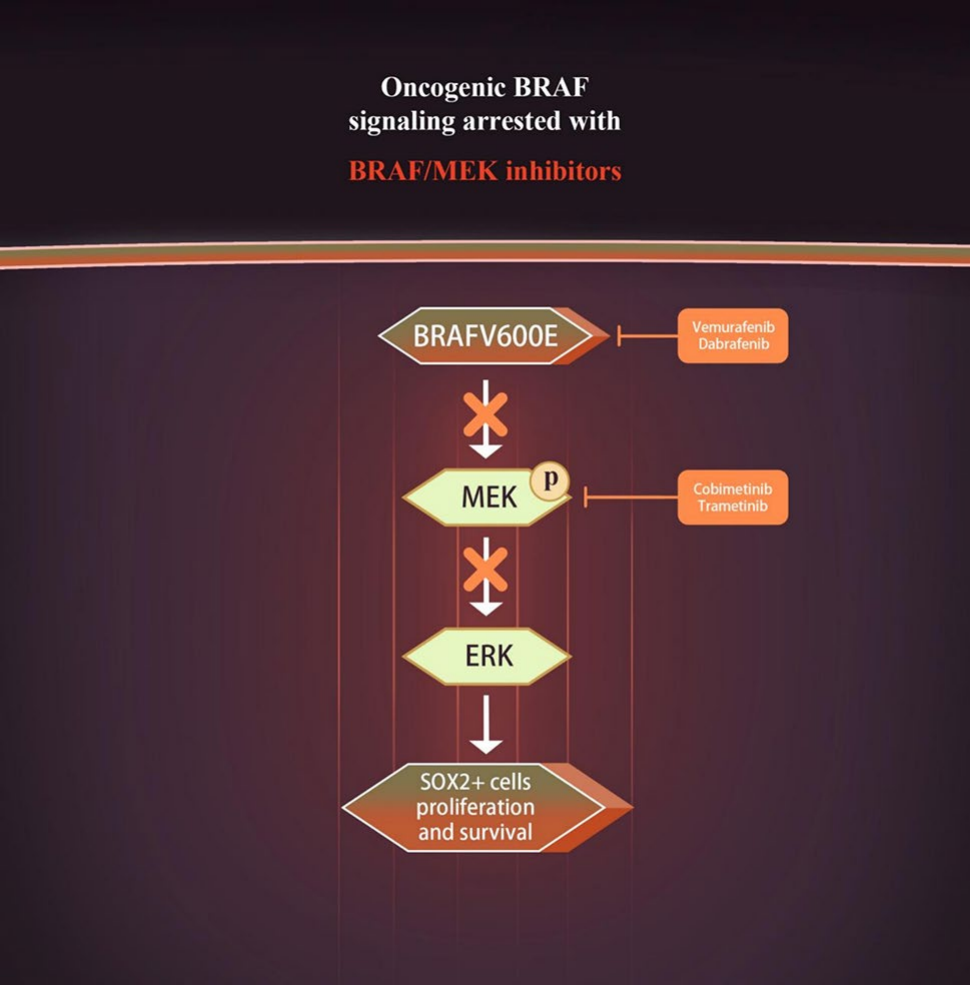
Inhibition of oncogenic BRAF signaling by BRAF/MEK inhibitors. Schematic illustration depicting the inhibition of oncogenic BRAFV600E signaling by specific inhibitors. The BRAFV600E mutation leads to sustained activation of the MAPK/ERK pathway, promoting the proliferation and survival of SOX2 + cells. BRAF inhibitors (Vemurafenib and Dabrafenib) specifically target and inhibit the mutant BRAF^V600E protein, while MEK inhibitors (Cobimetinib and Trametinib) inhibit the downstream effector molecule MEK. Combined use of these inhibitors effectively blocks aberrant signaling, reducing cell proliferation and survival. BRAFV600E, B-Raf proto-oncogene with V600E mutation; MEK, mitogen-activated protein kinase kinase; ERK, extracellular signal-regulated kinase; SOX2, SRY-box transcription factor 2

The advent of targeted therapy for PCP marks the beginning of a new era in its treatment. In recent years, nearly all PCP cases (almost 100%) have been found to harbor the BRAF V600E mutation, making the detection of this mutation an essential diagnostic criterion for PCP^7^. Based on this molecular mechanism and the use of BRAF/MEK inhibitors in other tumors harboring the BRAF V600E mutation [48–50], BRAF/MEK inhibitors have emerged as an effective treatment strategy for PCP. BRAF inhibitors (e.g., vemurafenib and dabrafenib) target and inhibit the BRAF V600E mutant protein, blocking its kinase activity and preventing downstream activation of MEK. By inhibiting BRAF, these drugs effectively block the MAPK/ERK signaling pathway, suppressing tumor cell proliferation and survival. In addition, MEK inhibitors (e.g., cobimetinib and trametinib) further inhibit MEK activation of ERK, thereby effectively blocking aberrant MAPK/ERK signaling downstream. This dual inhibition not only reduces the sustained activation of the signaling pathway but also effectively suppresses the proliferation and survival of SOX2 + tumor stem cells. By targeting the BRAF V600E mutation and blocking the MAPK/ERK signaling pathway, targeted therapy significantly reduces the proliferation of PCP tumor cells, slowing or even halting disease progression (Fig. 3).

In 2015, the first report demonstrated significant therapeutic efficacy using combined BRAF and MEK inhibition in a case of multiply recurrent papillary craniopharyngioma (PCP) [51, 52]. This study employed the BRAF inhibitor dabrafenib (150 mg, orally twice daily) and the MEK inhibitor trametinib (2 mg, orally twice daily). After 35 days of treatment, imaging revealed an 85% reduction in tumor volume. The residual tumor was subsequently surgically resected, and whole-exome sequencing of the specimen did not detect any mutations typically associated with resistance to MAPK pathway inhibition. Following this initial report, increasing cases of targeted therapy for PCP have been published (Table 1). As of August 31, 2024, a search in databases such as PubMed and Web of Science identified 16 studies on PCP-targeted therapy, collectively involving a total of 46 PCP patients, the initial cases were 33, and the recurrent cases were 13. The main therapeutic regimens included BRAF inhibitors (such as dabrafenib and vemurafenib) and MEK inhibitors (such as trametinib), administered either as monotherapy or in combination.

**Table 1:** Sixteen pieces of research literature on targeted treatment of papillary craniopharyngioma

References	Publication year	Treatment subjects	Treatment regimen	Treatment outcome	Analysis and discussion
Brastianos et al. [51, 52]	2015–2016	One 39-year-old male patient with recurrent PCP	Dabrafenib (150 mg, 2 times/day) + Trametinib (2 mg, 1 time/day)	Tumor volume reduced by 85%, Ki-67 proliferation index significantly decreased (from 22.1% to < 0.5%), significant clinical symptom improvement, no new symptoms appeared	This study strongly demonstrates the effectiveness of combined BRAF/MEK inhibitors in reducing tumor volume and proliferation, but lacks long-term follow-up data to confirm the durability of the response and does not explore long-term adverse effects after treatment
Aylwin et al. [53]	2015	One female patient with recurrent PCP	Vemurafenib	Significant tumor volume reduction, rapid tumor recurrence after treatment discontinuation, tumor volume reduced to near complete regression within 3 months of initial treatment	This study highlights the potential for rapid tumor recurrence after stopping treatment, suggesting the need for continuous treatment; however, the study lacks statistical analysis and has a small sample size, limiting the generalizability of the results
Roque and Odia [54]	2017	One 47-year-old female PCP patient	Dabrafenib (150 mg, 2 times/day) + Trametinib (2 mg, 1 time/day)	Significant tumor volume reduction, tumor reduced to one-third of the original volume within 2 months (from 3.2 to 1.6 cm), gradual neurological improvement, but residual permanent panhypopituitarism	This case shows promising treatment effects, and the observed permanent endocrine dysfunction emphasizes the need for close monitoring of side effects; however, the lack of a control group limits the assessment of relative treatment efficacy
Rostami et al. [55]	2017	One 65-year-old male patient with recurrent PCP	Dabrafenib + Trametinib	Tumor volume reduced by 91%, clinical symptoms improved	The rapid tumor reduction in this case supports the use of targeted therapy in recurrent PCP patients, but does not address long-term prognosis or potential resistance, requiring validation in larger controlled studies
Himes et al. [56]	2019	One 52-year-old male patient with recurrent PCP	Dabrafenib	Tumor volume reduced by more than 50%, no tumor progression occurred	This case demonstrates the effectiveness of Dabrafenib in controlling tumor growth, but does not provide long-term follow-up data to assess the durability of the response, and the lack of a control group also limits the generalizability of the study results
Juratli et al. [57]	2019	One PCP patient	Dabrafenib + Trametinib	Tumor volume nearly completely reduced (reduced by 85% to 90%), good treatment tolerance, no serious adverse reactions occurred	This study supports the use of combined BRAF/MEK inhibitor therapy, showing strong responsiveness in multiple cases; however, due to the lack of randomized controlled trial design, it is not possible to determine the difference in effects with other treatment methods, and future studies should continue to explore resistance mechanisms and long-term prognosis
Rao et al. [58]	2019	One 35-year-old male patient newly diagnosed with PCP	Dabrafenib (150 mg, 2 times/day)	Significant reduction in cystic components, clinical symptoms improved	This case emphasizes the potential of monotherapy in newly diagnosed PCP patients, but lacks comparative data with combination therapies, and the long-term safety of treatment remains unknown
Khaddour et al. [59]	2020	One 39-year-old male PCP patient	Dabrafenib (150 mg, 2 times/day) + Trametinib (2 mg, 1 time/day), followed by stereotactic radiosurgery	Tumor volume reduced by more than 70%, no recurrence signs within 18 months after radiation therapy	This case emphasizes the potential of neoadjuvant targeted therapy in reducing tumor volume for final treatment, but does not address the issue of resistance that may arise from long-term treatment, and lacks larger cohort studies to limit the strength of the conclusions
Chik et al. [60]	2021	One 37-year-old male PCP patient (onset in childhood)	Vemurafenib (960 mg, 2 times/day, continued for 40 months)	Tumor reduced by 55% within 6 weeks, dose reduction required due to elevated creatine kinase, tumor reduced again after full dose restoration	This case shows significant efficacy of Vemurafenib in childhood-onset papillary craniopharyngioma (although rapid tumor recurrence after drug withdrawal); long-term follow-up indicates the necessity of continuous treatment, but also raises concerns about long-term safety and resistance
Calvanese et al. [61]	2022	Two adult PCP patients	Dabrafenib + Trametinib	Significant tumor volume reduction, no tumor recurrence	The study results support the use of BRAF/MEK inhibitors in the treatment of adult PCP, but due to the lack of randomized controlled trial design, the strength of the conclusions is limited, and the potential for resistance or long-term adverse reactions also needs to be further explored in a larger patient population
Shah et al. [62]	2023	One 57-year-old female PCP patient	Concurrent radiotherapy and Dabrafenib + Trametinib	No tumor recurrence during 4 years of follow-up, improvement in right eye vision	This is the first reported case of concurrent radiotherapy and targeted therapy for PCP, showing promising results, and this method is worth further exploration; of course, larger studies are also needed to investigate long-term toxicity, especially toxicity related to the optic nerve
Lin et al. [63]	2023	One 59-year-old male patient suspected of PCP	Dabrafenib (reduced dose due to side effects)	Tumor volume significantly reduced within 19 days, tumor near complete remission after 6.5 months, tumor stable for 2.5 months after dose reduction	This case demonstrates the utility of BRAF inhibitors in PCP treatment, but also points out the need to adjust doses or implement personalized dosing strategies to balance efficacy and tolerability
Brastianos et al. [64]	2023	Sixteen untreated PCP patients	Vemurafenib + Cobimetinib	94% of patients achieved partial response or better within 4 months of treatment, median tumor volume reduction of 91%	The study provides strong evidence for the effectiveness of BRAF/MEK inhibitors in untreated PCP patients (although the small sample size and single-group design limit the generalizability of the results, and long-term follow-up is also needed to assess the durability of the response and potential late toxicity)
Losa et al. [65]	2024	One 75-year-old male patient with recurrent PCP	Dabrafenib + Trametinib	Initial rapid tumor reduction, recurrence observed after treatment discontinuation, tumor reduction upon resumption; second treatment maintained for 20 months, recurrence observed after 3 months of discontinuation; tumor reduced again upon reinitiation	This case emphasizes the effectiveness of BRAF/MEK inhibitors, but also reveals the challenges in managing recurrence after treatment withdrawal; the results emphasize the need for continuous treatment to prevent recurrence, thus raising discussions about long-term management strategies
De Alcubierre et al. [66]	2024	Sixteen PCP patients with BRAF V600E mutation	Dabrafenib (150 mg, twice daily) + Trametinib (1–2 mg, once daily)	94% of patients exhibited partial response or better, mean tumor volume reduction of 81.4%, good overall tolerability	This study demonstrates the effectiveness of BRAF/MEK inhibitors in treating PCPs, illustrating a substantial tumor reduction and acceptable safety profile. However, long-term follow-up and randomized trials are necessary to confirm these findings and explore resistance and long-term management strategies

Notably, combination therapy was more frequently utilized. The dual inhibition mechanism has demonstrated remarkable efficacy in suppressing tumor cell proliferation. Based on current data, the majority of patients achieved partial or better objective responses following treatment, with a median tumor volume reduction ranging from 70 to 91%. Most patients exhibiting sustained clinical and radiological improvement.

Common adverse effects associated with this therapy included rash, fever, and fatigue. A small percentage of patients (approximately 5%) experienced severe adverse events, such as grade 4 hyperglycemia and elevated creatine kinase levels. Although the overall tolerability of targeted therapy was favorable, some patients experienced tumor recurrence after discontinuing treatment, suggesting that continuous therapy may be necessary to maintain clinical benefit in certain cases. In addition, some patients discontinued treatment due to drug toxicity or intolerance, highlighting the need for careful management of treatment-related side effects.

In conclusion, while targeted therapies such as BRAF/MEK inhibitors show significant promise in treating PCP, challenges related to recurrence, long-term safety, and resistance remain. Larger, controlled studies and longer follow-up are essential to further evaluate the efficacy and safety of these therapies, as well as to develop strategies for managing potential relapse and long-term adverse effects.

One of the most notable studies in this field is a single-arm, Phase II clinical trial conducted by Brastianos et al. [64], which evaluated the efficacy of combined BRAF-MEK inhibition in patients with BRAF V600E-positive PCP. The study enrolled patients with measurable PCP who had not undergone radiotherapy and who were positive for the BRAF mutation. Sixteen patients received a combination of the BRAF inhibitor vemurafenib and the MEK inhibitor cobimetinib in 28-day treatment cycles. Of the 16 patients, 15 (94%) exhibited a durable partial response or better, with a median tumor volume reduction of 91% (range, 68–99%). The median follow-up duration was 22 months, and the median number of treatment cycles was eight. Progression-free survival (PFS) was 87% at 12 months and decreased to 58% at 24 months. Three patients experienced disease progression during the follow-up period, but no deaths were reported. One patient discontinued treatment due to toxicity after eight days, and 12 patients experienced at least one grade 3 adverse event potentially related to treatment, while two patients had grade 4 adverse events. This small, single-arm study demonstrated that 15 patients achieved a partial response or better with vemurafenib–cobimetinib combination therapy, suggesting that BRAF–MEK inhibitors may represent an effective treatment option for PCP patients who have not undergone radiotherapy. The study also explored the potential for detecting circulating BRAF V600E mutation DNA in plasma samples, which could provide a noninvasive method for disease monitoring. Although the BRAF–MEK combination inhibition therapy has been highly praised [67], the study has its limitations. It was a single-arm study without a control group and had a small sample size, which limits the generalizability of the results. Additionally, the study highlighted the need for further research to determine the optimal treatment duration and manage the toxicity of long-term therapy.

Despite the encouraging results of these studies, several limitations and potential biases remain:

1. **Small sample size and heterogeneity**: Most of the available studies are case reports or small sample studies, with sample sizes ranging from 1 to 16 patients per study, lacking large-scale randomized controlled trials. This limits the statistical significance and generalizability of the findings.
2. **Diverse treatment protocols**: The use of varying treatment regimens, including monotherapy, combination therapy, and combinations of radiotherapy with targeted therapy, adds complexity to the results and makes it difficult to identify the optimal therapeutic approach.
3. **Lack of long-term follow-up data**: Although many studies report short-term outcomes, long-term follow-up data are lacking, especially for recurrent PCP patients. Long-term data are critical for assessing the durability of treatment efficacy and safety. Moreover, the most reports are case-based, raising the possibility of selection bias—successful cases are more likely to be reported, while cases with treatment failure or significant adverse events may be underreported. Without control groups, it is difficult to assess the true efficacy of BRAF/MEK inhibitors compared to other treatment modalities such as surgery or radiotherapy.

BRAF/MEK inhibitors have shown significant efficacy in treating recurrent PCP, but due to the small sample sizes and high heterogeneity of the studies, the results may not be fully applicable to all PCP patients, especially those with complex or comorbid conditions. Several reports indicate tumor recurrence after treatment cessation, suggesting that continuous therapy may be essential for maintaining therapeutic efficacy. Future studies should focus on determining the optimal treatment duration and managing the risk of relapse after discontinuation. Although BRAF/MEK inhibitors are generally well-tolerated, some patients have experienced severe adverse events, and tumor recurrence has been reported after drug cessation, raising concerns about long-term safety in broader patient populations.

In summary, BRAF/MEK inhibitors have shown significant efficacy in the treatment of recurrent PCP, particularly in cases of recurrent disease. However, the limited sample sizes, study design limitations, and potential selection biases highlight the need for further validation of these results. Future research should focus on large-scale randomized controlled trials and long-term follow-up data to provide more statistically significant and clinically relevant evidence. Additionally, studies should explore the optimal duration of treatment and strategies for managing recurrence after treatment cessation. Although BRAF/MEK inhibitors have shown good tolerability, severe adverse events in some patients underscore the need for careful consideration of safety, especially in long-term use.

## Management of long-term side effects and potential resistance in targeted therapy

Clinical experience has shown that long-term use of targeted therapy drugs such as vemurafenib may lead to several side effects, including dermatologic toxicity, liver damage, and cardiovascular events [49]. To ensure treatment safety, regular monitoring and assessment of patients undergoing targeted therapy for PCP are necessary, with adjustments to the therapeutic regimen when needed [55]. Symptomatic treatment, including temporary discontinuation of therapy, may help alleviate adverse effects. For example, skin rash and diarrhea associated with BRAF inhibitors in PCP patients can be managed through skin care and dietary adjustments [53].

Although no definitive evidence of resistance to BRAF/MEK inhibitors in PCP has been reported, the frequent recurrences after discontinuation prompt consideration of how to prevent and overcome resistance. Several strategies have been suggested based on current literature:

1. **Optimizing treatment protocols**: Determining the optimal treatment duration and combination therapy strategies to prevent resistance [65].
2. **Developing new drugs**: Research into new targeted therapies is ongoing, with studies investigating potential therapeutic targets such as PD-L1, CTNNB1, and EGFR [68]. Strategies used to overcome resistance in BRAF–mutant melanoma, such as the development of next-generation targeted drugs and combination therapies to inhibit multiple signaling pathways, could be adapted for PCP [50].
3. **Monitoring and adjusting therapy**: Close monitoring of tumor response during treatment using multiple blood tests for circulating BRAF V600E mutation levels could provide real-time data on the tumor’s response to therapy. No resistance mutations have been detected in PCP after targeted therapy thus far [51].
4. **Combination therapy with other modalities**: Combining targeted therapy with other treatments such as radiotherapy or surgery is another important area of research. For instance, in one case of recurrent PCP, a patient undergoing intensity-modulated radiation therapy (IMRT) planned for a total dose of 5400 cGy had their treatment paused after 2160 cGy due to vision deterioration and cyst enlargement. The patient then underwent debulking surgery and endoscopic transnasal fenestration to alleviate symptoms. Four months later, radiotherapy resumed, and the cumulative dose reached 5940 cGy. The patient also received a cycle of dabrafenib and trametinib, which yielded a good clinical response, with improved vision and MRI showing controlled tumor growth. No recurrence was observed during a four-year follow-up [62].

## Discovery and targeted therapy of pediatric papillary craniopharyngioma (PPCP)

Although PCP is primarily seen in adults, reports of pediatric papillary craniopharyngioma (PPCP) have surfaced. The earliest documented PPCP case dates back to 2002, when Zhang identified two PCP cases out of 189 pediatric craniopharyngiomas based on histopathological HE staining, with the patients’ ages ranging from 1 to 15 years, though their exact ages were not provided [69]. At that time, the BRAF V600E mutation had not yet been reported in PCP, so no mutation testing was conducted. In 2016, Cheng reported 16 cases of papillary craniopharyngioma out of 92 pediatric craniopharyngiomas in patients aged 1 to 16 years, but this report lacks sufficient supporting data and is questionable [70]. Tariq et al. [71] described a clinicopathological study of 13 PCP cases, including two pediatric cases. Unfortunately, all these early reports were based on histopathological HE staining without BRAF V600E testing, making it difficult to assess whether misdiagnosis occurred. It was not until 2017 that Chik reported a case of pediatric PCP initially diagnosed histopathologically without BRAF V600E testing, but upon recurrence in adulthood, the mutation was detected, and the patient responded well to BRAF inhibitor therapy [60]. In 2018, Schlaffer reported the first confirmed pediatric PCP case with BRAF V600E testing in a 6-year-old, asserting that PCP can occur in children and suggesting that PPCP may originate from Rathke's cleft cysts [72]. In 2019, Borrill documented a 4-year-old with BRAF V600E-positive PPCP, proposing targeted therapy with BRAF inhibitors as a potential alternative to stereotactic radiation therapy, reducing the risk of radiotherapy-related developmental issues in children [73]. In 2021, Takagi reported a 14-year-old with BRAF V600E-positive PPCP, characterized by an irregularly enhancing cystic mass with an associated abscess [14]. Zhao et al. [13] conducted the first study to define the imaging and histopathological features of five BRAF V600E-positive PPCP cases. Although this confirmed the existence of PPCP and clarified some of its clinical features, BRAF-targeted therapy in this cohort failed, with surgical resection ultimately achieving cure. Unfortunately, the study did not explore the reasons for the failure of targeted therapy in these cases.

## Comprehensive treatment strategies for PCP and future prospects

This review has summarized the molecular characteristics of papillary craniopharyngioma (PCP) and the latest advances in various therapeutic approaches (Table 2). Although targeted therapy has shown significant potential in the management of PCP, optimizing treatment regimens remains critical. This includes determining appropriate drug dosages, treatment durations, and combination therapy strategies. Future research should prioritize the discovery and validation of new molecular targets, understanding how the tumor immune microenvironment influences treatment response, and developing more precise, individualized treatment plans to improve overall survival and quality of life for patients.

**Table 2:** Treatment modalities and their pros/cons

Treatment modality	Pros	Cons
Surgical resection	Potential for complete tumor removal	High risk of damage to surrounding structures
	Immediate resolution of mass effect	Potential for significant postoperative complications
Radiation therapy	Noninvasive	Potential long-term effects on healthy tissue
	Can target residual tumors post surgery	Requires multiple sessions
Stereotactic radiosurgery	Precise targeting minimizes damage to surrounding tissue	Limited to small tumor volumes
	Effective for small residual tumors	Risk of radiation injury if higher doses are used
BRAF/MEK inhibitors	Targeted action on BRAF V600E mutation	Possible side effects such as skin rash, fever, fatigue
	Shows promising reduction in tumor size	Continuous therapy may be needed to maintain remission
Intracystic chemotherapy	Effective for cystic component reduction	Significant neurotoxicity risk
	Can facilitate surgical resection	Potential for leakage into cerebrospinal fluid leading to severe complications

**Fig. 4:**
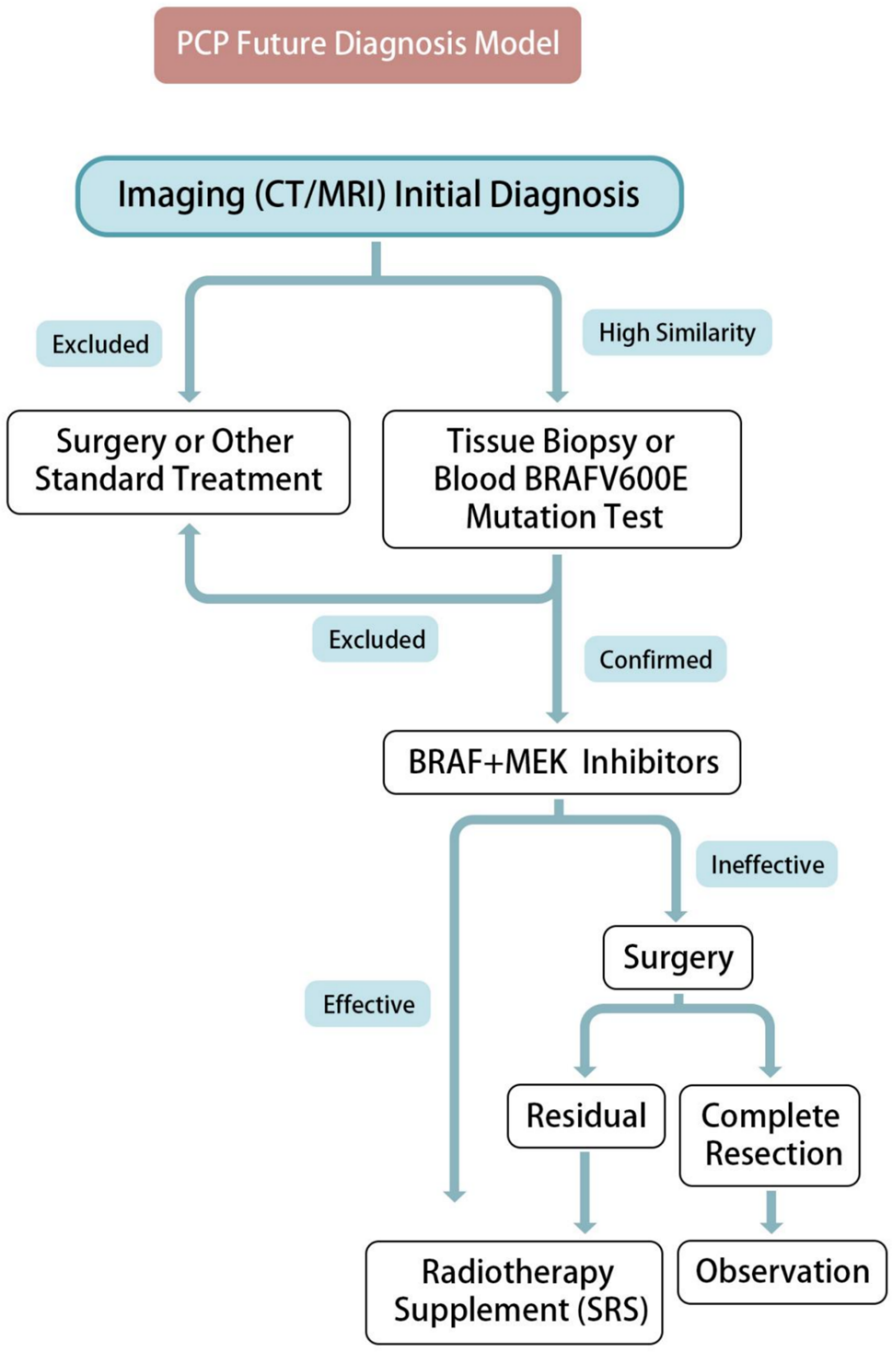
Future therapeutic model for PCP. MRI, magnetic resonance imaging; CT, computed tomography; BRAFV600E, B-Raf proto-oncogene V600E mutation; SRS, stereotactic radiosurgery

The future treatment model for PCP may begin with imaging (CT/MRI) to establish a preliminary diagnosis, guiding the appropriate treatment pathway. For cases with high suspicion, stereotactic histological biopsy or detection of circulating BRAF V600E mutations in blood samples can provide key information for utilizing BRAF and MEK inhibitors, a promising targeted therapy. However, this approach may vary between individuals, and some patients may require additional surgical intervention, stereotactic radiosurgery (SRS), or close monitoring (Fig. 4). It is also important to consider differences between adult and pediatric cases.

This comprehensive treatment strategy underscores the importance of precision medicine in the treatment of PCP. Future research should focus on optimizing treatment sequencing, dosage, and duration, identifying which patient populations are most likely to benefit from novel targeted therapies, and exploring how these therapies interact with the tumor microenvironment. Furthermore, the development of more tailored combination therapies based on individual patient characteristics will be crucial for improving treatment outcomes. As part of an integrated treatment strategy, targeted therapy is expected to drive a significant shift in the current treatment paradigm for PCP. With further refinement, these approaches hold promise for providing neurosurgeons with more effective tools for managing this historically challenging tumor.

## Conclusion

This review has elucidated the molecular characteristics of papillary craniopharyngioma (PCP) and explored core mechanisms driving tumorigenesis, particularly emphasizing the role of the BRAF V600E mutation. Recent clinical advancements with targeted therapies, notably BRAF and MEK inhibitors, mark a significant shift in the treatment landscape for PCP. The integration of comprehensive treatment strategies, which include surgery, radiation, and targeted therapy, offers a promising avenue for personalized treatment approaches. However, the current studies are limited by small sample sizes and significant heterogeneity, underscoring the need for large-scale randomized controlled trials to validate these therapeutic approaches' long-term efficacy and safety. Future research should focus on optimizing treatment sequencing, dosage, and duration, although exploring how the tumor microenvironment influences therapeutic response. The development of more precise combination therapies tailored to individual patient characteristics will be crucial for improving overall survival and quality of life. Ongoing molecular biology research holds the potential to drive innovation in PCP management strategies.

## Data Availability

No datasets were generated or analyzed during the current study.
